# Synthesis and Characterisation of Monolacunary Keggin Monovanado-deca-tungstophosphate and Its Complexes with Transition Metal Cations

**DOI:** 10.3390/ma16020827

**Published:** 2023-01-14

**Authors:** Anda Ioana Gratiela Petrehele, Narcis Duteanu, Mona Claudia Morgovan, Sanda Monica Filip, Stefania Ciocan, Eleonora Marian

**Affiliations:** 1 Department of Chemistry, Faculty of Informatics and Sciences, University of Oradea, 1 University Street, 410087 Oradea, Romania; 2 Faculty of Industrial Chemistry and Environmental Engineering, Politehnica University of Timisoara, 2 Victoriei Square, 300006 Timisoara, Romania; 3 Department of Physics, Faculty of Informatics and Sciences, University of Oradea, 1 University Street, 410087 Oradea, Romania; 4 Department of Pharmacy, Faculty of Medicine and Pharmacy, University of Oradea, 29 Jiga Street, 410028 Oradea, Romania

**Keywords:** polyoxometalates Keggin, mixed addenda, tungsten, vanadium, spectroscopy

## Abstract

Five new complexes with metal cations (Mn^2+^, Fe^3+^, Co^2+^, Ni^2+^, and Cu^2+^) of monolacunary Keggin monovanado-deca-tungstophosphate, K_8_[PVW_10_O_39_]·15H_2_O, have been synthesised. The molar ratio of the combination between metal cations and K_8_[PVW_10_O_39_]·15H_2_O has been established to be 1:1, and its general molecular formulas were found to be: K_n_[MPVW_10_O_39_(H_2_O)]·xH_2_O (*n* = 5 for M = Fe^3+^ and *n* = 6 for M = Mn^2+^, Co^2+^, Ni^2+^, and Cu^2+^). Optimal conditions for complexes’ synthesis (pH, temperature, and reaction time) have been determined. The characterisation of K_8_[PVW_10_O_39_]·15H_2_O and of its compounds K_n_[MPVW_10_O_39_(H_2_O)]·xH_2_O have been performed using AAS, TG-DTA-DTG, UV-VIS, IR, Raman, and powder XRD methods. In UV spectra, two maximums of absorption were obtained, at 200 and 250 nm, characteristic of Keggin polycondensate compounds. The coordination of cations Ni^2+^, Co^2+^, and Cu^2+^ through oxygen atoms from K_8_[PVW_10_O_39_]·15H_2_O in an octahedron system has been reflected with VIS spectroscopy. All these methods have proved the compositions and structures of K_8_[PVW_10_O_39_]·15H_2_O and K_n_[MPVW_10_O_39_(H_2_O)]·xH_2_O, their similarity with other vanadotungstophosphates, and their achievements in the Keggin class. Additionally, all analysis methods have shown an increase in the degree of structural symmetry and the thermal stability of a polyoxoanion complex after attaching metal cations compared to the monolacunary, K_8_[PVW_10_O_39_]·15H_2_O.

## 1. Introduction

Polyoxometalates, Keggin type with a complete structure have the general formula [XM_12_O_40_]^n-^ and are a class of polycondensation inorganic compounds formed by twelve units of MO_6_ (where M is Mo^6+^, W^6+^, V^5+^, Nb^5+^, or Ta^5+^, named addenda atoms) binding together through commune corners and edges. Units of MO_6_ coordinate around a tetrahedral unit, XO_4_, where X can be almost any element of the periodic system [1]. Three MO_6_ octahedrons are connected together by common edges in four M_3_O_13_ units, which in turn are connected by common corners. Oxygen atoms can be distinguished by the nature of the connection in Oi (oxygen shared between each of the three octahedrons of the M_3_O_13_ unit and XO_4_ group) in X-Oi-M bonds; Oe (oxygen shared between the MO_6_ octahedron of the same M_3_O_13_ unit) in M-Oe-M bonds; Oc (oxygen shared between the MO_6_ octahedron of a different M_3_O_13_ unit) in M-Oc-M bonds; and Ot, unshared oxygen from each MO_6_ octahedron (terminal oxygen) in M = Ot bonds. Keggin polyoxoanions [XM_12_O_40_]^n-^ may lose one, two, or three units of MO_6_ to transform into a lacunary form, more active than the complete structure, so that they can act as chelating ligands of cation metals. All the bonds between heteroatoms X and addenda atoms M in polyoxoanions are carried out by oxygen atoms, which may become electron pair donors for external metal cations. The ability of polyoxometalates to engage a wide variety of metal atoms leads to an increase in the synthesis of new compounds [1,2,3]. Moreover, special chemical and physical properties of polyoxometalates facilitate their use in various fields such as chemical catalysis [4,5,6,7], analytical chemistry [8], biochemistry [9], and medicine [10]. The authors of this article have already published an application on monovanado-deca-tungstophosphate polyoxometalates, K_8_[PVW_10_O_39_]·15H_2_O, and its complexes K_n_[MPVW_10_O_39_(H_2_O)]·xH_2_O (*n* = 5 for M = Fe^3+^ and *n* = 6 for M = Mn^2+^, Co^2+^, Ni^2+^, and Cu^2+^) on *Triticale* seed germination, where the involvement of these polyoxometalates in the growth of *Triticale* seedlings during the first days after germination and the possibility to use these compounds as fertilizers were presented and discussed [11].

This paper is focused on the synthesis and structural analysis of polyoxometalates K_8_[PVW_10_O_39_]·15H_2_O and K_n_[MPVW_10_O_39_(H_2_O)]·xH_2_O (*n* = 5 for M = Fe^3+^ and *n* = 6 for M = Mn^2+^, Co^2+^, Ni^2+^, and Cu^2+^). In the first part, the synthesis of monolacunary Keggin, K_8_[PVW_10_O_39_]·15H_2_O was achieved in two steps, according to reactions (1) and (2) [12].

H_3_PO_4_ + 9 Na_2_WO_4_ + 10 H_3_C-COOH → Na_8_[HPW_9_O_34_] + 10 H_3_C-COONa + 6 H_2_O (1)

Na_8_[HPW_9_O_34_] + Na_2_WO_4_ + NaVO_3_ + 3 HCl + 8 KCl → K_8_[PVW_10_O_39_] + 11 NaCl + 2 H_2_O (2)

In the second part, the stoichiometry of reactions between monolacunary polyoxometalate, K_8_[PVW_10_O_39_]·15H_2_O and metal transition cations M (where M is Mn^2+^, Fe^3+^, Co^2+^, Ni^2+^, and Cu^2+^) in the synthesis of K_n_[MPVW_10_O_39_(H_2_O)]·xH_2_O complexes was determined. The result of this study was that the coordination of monolacunary polyoxometalate, K_8_[PVW_10_O_39_]·15H_2_O to the metal cation M took place according to the following reaction scheme:

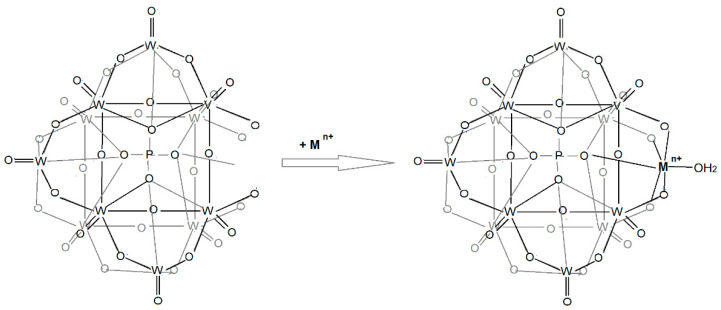


**In addition, the optimal reaction conditions for the formation of K_n_[MPVW_10_O_39_(H_2_O)]·xH_2_O complexes, pH, reaction time, and temperature were determined.** Then, K_n_[MPVW_10_O_39_(H_2_O)]·xH_2_O complexes were prepared and characterised using AAS, thermogravimetry, FT-IR, FT-Raman, UV-VIS, and XRD methods.

## 2. Materials and Methods

All reagents were purchased from commercial sources and used as received. Distilled water was used in all procedures. Conductometer and pH meter analyses have been performed using a Multi 720 Inolab WTW series. Elemental analysis of P, Mo, V, Mn, Fe, Co, Ni, and Cu was performed with a Varian ASA 220 type spectrophotometer (Palo Alto, CA, USA). Potassium has been determined with an Eppendorf flame photometer (Hamburg, Germany). TG-DTA-DTG curves were used for the determination of the new synthesized compounds’ thermal stabilities, and further, for the determination of the number of water molecules in air on a Paulik-Erdely OD-103 (Budapest, Hungary) derivatograph (20–800 °C) at 5 °C min^−1^. FT-IR spectra were recorded in the 4000–400 cm^−1^ range on a Biorad FTS 60A spectrophotometer (ThermoFisher Scientific, Waltham, MA, USA) using KBr pellets. Raman spectroscopy was performed on solid powders at room temperature, using a DILOR OMARS 89 Raman spectrophotometer (Thousand Oaks, CA, USA) (λe = 1064 nm). UV-VIS spectra were recorded in the range 190–1100 nm on a T-60 UV-VIS model spectrophotometer (ThermoFisher Scientific, Waltham, MA, USA). Powder XRD patterns were obtained using a Bruker D8 Advance powder diffractometer (Billerica, MA, USA) at 40 kV and 40 mA, equipped with an incident beam Ge 111 monochromator using CuKα_1_ radiation (λ = 1.540598 Å). The patterns have been indexed using the Dicvol method. Accelrys Software Inc., Materials Studio (Cambridge, UK), Release 5.5, 2010 was used to assess the degree of crystallinity. The experimental density was determined using a pycnometer from each polyoxometalate dissolved in acetone.

### 2.1. Synthesis of K_8_[PVW_10_O_39_]·15H_2_O (L)

The trilacunary polyoxometalate, α, A-Na_8_[HPW_9_O_34_]·11H_2_O, was prepared according to the method from the literature [12]. In a beaker, a mixture of 1.95 g (10 mmol) of NaVO_3_·4H_2_O and 3.3 g (10 mmol) of Na_2_WO_4_·2H_2_O were dissolved in 50 mL of distilled water and 26.13 g (10 mmol) of α, and A-Na_8_[HPW_9_O_34_]·11H_2_O was added under vigorous stirring. The pH was adjusted to 5.5 with the addition of a 3 M HCl solution. The insoluble materials were removed by filtration under suction, and 7.45 g (100 mmol) of KCl was added to the red filtrate. Orange crystals started to develop from the solution stored at 5 °C for 30 min. Crystals of **L** were collected and washed with 20 mL of deionised water, 20 mL of ethanol, and 20 mL of diethyl ether. The resulting mixture was dried in a desiccator. The yield was 15.00 g (47.97%). UV: 206.5; 249 (nm); IR: 3415 vb, 1629 b, 1072 m, 1013 w, 943 vs, 880 sh, 827 s, 807 m, 752 s, 615, 594, and 508 (cm^−1^); Raman: 960, 889, 505, 376, 229, and 150 (cm^−1^); Analytically calculated cd. (found) (M = 3127.4051): K, 10.00 (9.75); P, 0.99 (1.00); V, 1.63 (1.50); W, 58.79 (58.5); and H_2_O, 8.63 (8.69)%.

### 2.2. Determination of Optimal Reaction Conditions for the Synthesis of Complexes ***1–5***

**Figure 1:**
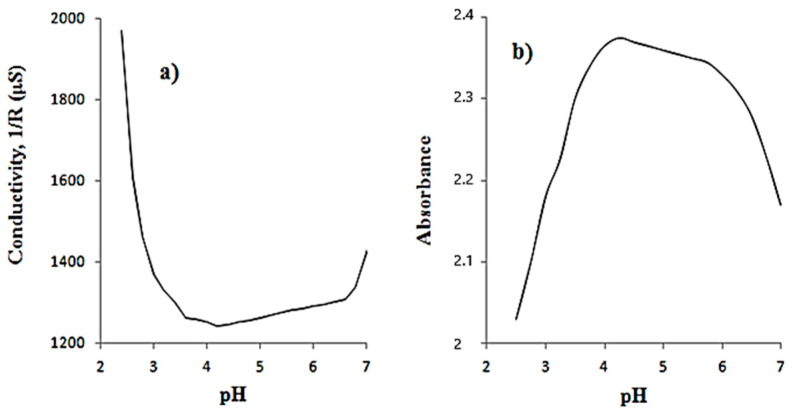
Determination of the stability of the pH range for **L** using (**a**) a conductometric method and (**b**) a spectrometric method at 206.5 nm.

Conductometric and spectrophotometric methods were used to determine the pH range where monolacunary L is stable. For the conductometric method, a 5.0 mM L solution (0.469 g; 0.15 mmol) was used and adjusted to pH 2.0 by the addition of 0.1 N HCl and brought to a pH of 7.0, gradually, using a 0.1 N KOH solution. Conductivities were measured and are shown in Figure 1a. For the spectrophotometric method, 0.01 mM of L was prepared in a 50 mL flask, and the pH was adjusted to the range 2.5–7.5, by adding 0.1 N HCl or 0.1 N KOH solutions. The absorbance of L solutions was measured at 206.5 nm and is shown in Figure 1b.

**Figure 2:**
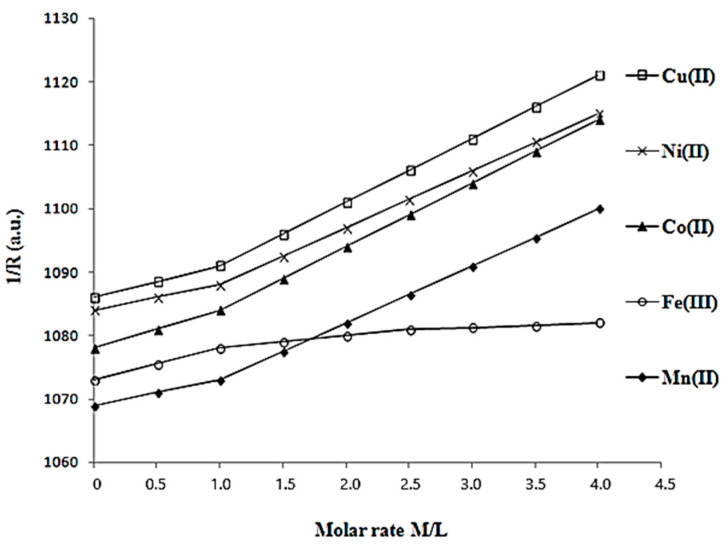
Determination of the molar combination ratio between monolacunary ligand **L** and metal cations M (Mn^2+^, Fe^3+^, Co^2+^, Ni^2+^, and Cu^2+^) in a buffer solution at pH 5.0 using the conductometric method.

The determination of the molar rate between **L** and metal cations M was performed using a conductometric method. In a beaker, a 1 mL solution of 5 mM of **L** (5·10^−3^ mmol) and a 4 mL buffer solution (acetic acid and sodium acetate) were added, corresponding to a pH of 5.0. The solution volume was adjusted to 30 mL using distilled water and a 10 mM MnCl_2_ solution was added in 0.25 mL amounts to achieve different M/L rates. The experiment and recording data were performed in the same way using 10 mM of FeCl_3_, CoCl_2_, and NiCl_2_, respectively. CuCl_2_ solutions were used instead of 10 mM MnCl_2_. The variation of conductivities from different M/L rates for all the experiments were recorded and are shown in Figure 2.

Optimal reaction conditions (pH, temperature, and reaction time) were determined using the colorimetric method. Measurements of absorbance were made only at a wavelength when metal cation complexes had a significant absorbance, while monolacunary ligand **L** and aquo complexes of cations had insignificant absorbances. Based on the criteria mentioned above, the measurement of the absorbance for the prepared samples was performed at 800 nm for Ni^2+^ and Cu^2+^ and at 870 nm for Fe^3+^, respectively, and at 1100 nm for Mn^2+^ and Co^2+^.

**Figure 3:**
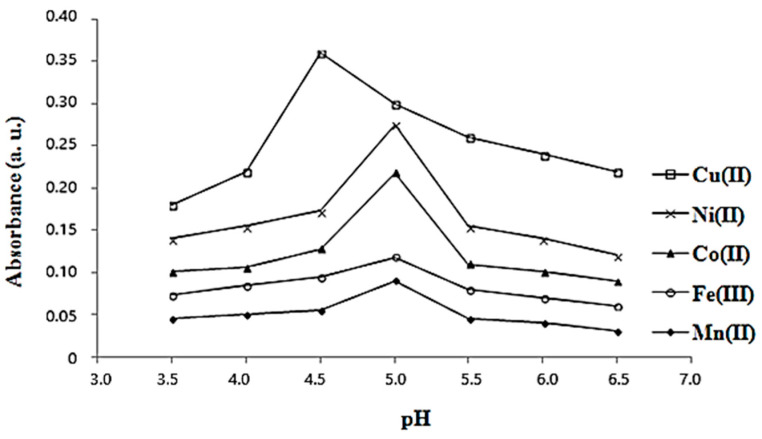
Determination of the optimal pH in synthesis for the complexes between ligand **L** and metal cation M (Mn^2+^, Fe^3+^, Co^2+^, Ni^2+^, and Cu^2+^) using a spectrophotometric method.

In a 50 mL flask, 20 mL of **L** and 20 mL of MnCl_2_ with the same concentration (5·10^−3^ M) were mixed, and the pH was adjusted from 3.5 to 6.5 with 0.1 N HCl and 5% KHCO_3_ solutions, respectively. The method was repeated for each metal cation solution mentioned previously in the molar rate study, instead of MnCl_2_. These reaction mixtures were used in the following studies. All measurements of absorbencies were undertaken at room temperature 30 min after preparation. The variation in absorbance at different pHs in the range 3.5–6.5 for each reaction mixture between solutions of metal cations and **L** was recorded in Figure 3.

**Figure 4:**
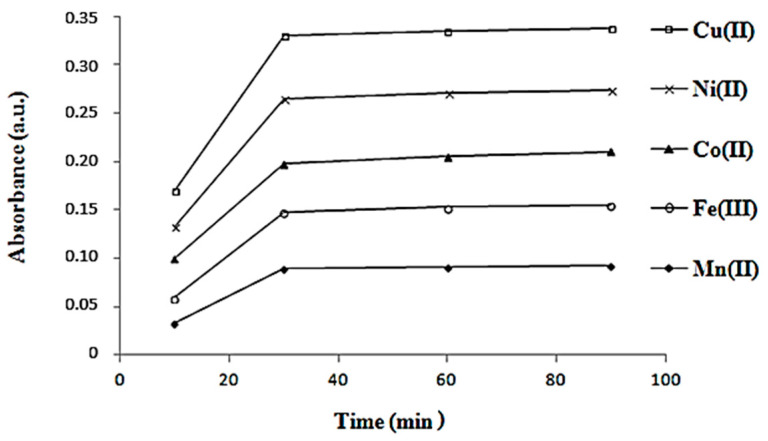
Determination of the optimal reaction time for the synthesis of complexes between ligand **L** and metal cation M (Mn^2+^, Fe^3+^, Co^2+^, Ni^2+^, and Cu^2+^) using a spectrophotometric method.

To determine the optimal reaction time, the absorbance of the mixtures prepared above was read after 10, 30, 60, and 90 min (Figure 4). The measurements were performed at room temperature under the optimal pH conditions already found for each metal cation, M.

**Figure 5:**
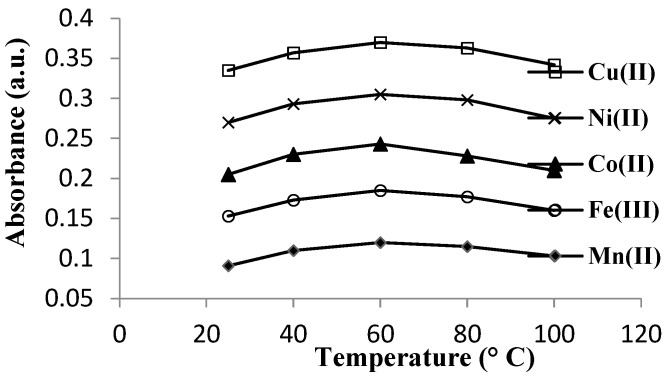
Determination of the optimal temperature for the synthesis of complexes between ligand **L** and metal cation M (Mn^2+^, Fe^3+^, Co^2+^, Ni^2+^, and Cu^2+^) using a spectrophotometric method.

To determine the optimal synthesis temperature, the absorbance of the reaction mixtures were performed at different temperatures: 25, 40, 60, 80, and 100 °C. Measurements was measured after an optimal reaction time and in optimal pH conditions ascertained from previous experiments. Figure 5 shows a plot of the curves of absorbance variation with temperature.

### 2.3. Synthesis of K_6_[MnPVW_10_O_39_(H_2_O)]·14H_2_O (***1***)

In a beaker containing 12.5 g (4.00 mmol) of **L** dissolved in distilled water, a solution resulting from the dissolution of 0.79 g (4.00 mmol) of MnCl_2_∙4H_2_O in a minimum quantity of water was gradually added. The pH of the solution was adjusted to 5.0 by adding dropp by drop solutions of HCl (0.1 M) and KHCO_3_ (5%). The mixture was kept at a temperature of 60 °C for 30 min. Any insoluble material was removed by filtration under suction, and 2.5 g (33.53 mmol) of KCl powder was added to the filtrate. The resulting red solution was kept in the fridge, at a temperature of 5 °C, for 2–3 days. After 3 days, red-orange crystals of **1** were filtered and then washed with ethanol. The crystals were kept for two days at room temperature in desiccators. Crystals of **1** were recrystallized from deionised water at a pH of 5.0 at room temperature. The yield was 7.34 g (59.1%). UV: 199, 251.5 (nm); IR: 3441 vb, 1617 b, 1082 m, 1058 m, 957 vs, 881 m, 792 s, 700 sh, 593, and 505 (cm^−1^); Raman: 987, 890, 519, 229, and 156 (cm^−1^); Analytically calculated (found) (M = 3104.1471): K, 7.56 (7.51); Mn, 1.77 (1.73); P, 1.00 (0.95); V, 1.64 (1.61); W, 59.23 (59.35); and H_2_O, 8.70 (8.78)%.

### 2.4. Synthesis of K_5_[FePVW_10_O_39_(H_2_O)]·11H_2_O (***2***)

For the synthetic procedure of **2**, the same steps as the synthesis of **1** were followed, as described before. A quantity of 12.5 g (4.00 mmol) of **L** was used and 0.65 g (4.00 mmol) of FeCl_3_ instead of MnCl_2_·4H_2_O. The addition of 2.5 g (33.53 mmol) of KCl to the solution led to orange crystals of **2**. The crystals were filtered off and dried in desiccators. The yield was 3.86 g (32.05%). UV: 204, 250 (nm); IR: 3444 vb, 2921, 2851, 1624 b, 1087 m, 1060 m, 961 vs, 883 m, 795 s, 595, and 505 (cm^−1^); Raman: 995, 890, 517, 223, and 155 (cm^−1^); Analytically calculated (found) (M = 3011.9122): Fe, 1.85 (1.75); K, 6.49 (6.26); P, 1.03 (1.00); V, 1.69 (1.62); W, 61.04 (61.10); and H_2_O, 7.17 (7.45)%.

### 2.5. Synthesis of K_6_[CoPVW_10_O_39_(H_2_O)]·21H_2_O (***3***)

The synthesis of **3** was performed using the same mode as **1**, adding 0.95 g (4.00 mmol) of CoCl_2_·6H_2_O instead of MnCl_2_·4H_2_O at 12.5 g (4.00 mmol). Red-orange crystals of **3** were formed after the addition of 2.5 g (33.53 mmol) of KCl in solution. The resulting mixture was then filtered and dried in desiccators. The yield was 8.07 g (62.38%). UV: 203, 251 (nm); IR: 3445 vb, 1617 b, 1077 m, 1058 m, 1051 m, 959 vs, 889 m, 802 s, 750 sh, 705 sh, 592, and 509 (cm^−1^); Raman: 989, 974, 890, 518, 229, and 154 (cm^−1^); Analytically calculated (found) (M = 3234.2492): Co, 1.82 (1.78); K, 7.25 (7.15); P, 0.96 (0.93); V, 1.58 (1.50); W, 56.84 (57.00); and H_2_O, 12.24 (12.72)%.

### 2.6. Synthesis of K_6_[NiPVW_10_O_39_(H_2_O)]·19H_2_O (***4***)

The synthesis method used in the preparation of complex **1** was followed for the preparation of complex **4**, using a mixture of 12.5 g (4.00 mmol) of **L** and 0.95 g (4.00 mmol) of NiCl_2_·6H_2_O. In solution, 2.5 g (33.53 mmol) of KCl was added, and red-orange crystals of **4** were formed after three days. The red-orange crystals of **4** were recrystallized in deionised water at room temperature. The yield was 8.65 g (67.63%). UV: 201, 252 (nm); IR: 3440 vb, 1617 b, 1080 sh, 1059 m, 958 vs, 883 m, 792 s, 700 sh, 592, and 507 (cm^−1^); Raman: 991, 980, 890, 517, 235, and 155 (cm^−1^); Analytically calculated (found) (M = 3197.9755): K, 7.34 (7.30); Ni, 1.84 (1.80); P, 0.97 (0.95); V, 1.59 (1.58); W, 57.49 (57.53); and H_2_O, 11.26 (11.21)%.

### 2.7. Synthesis of K_6_[CuPVW_10_O_39_(H_2_O)]·14H_2_O (***5***)

The synthetic procedure mentioned above was followed for the synthesis of **5**, using 0.68 g (4.00 mmol) of CuCl_2_·2H_2_O instead of MnCl_2_·4H_2_O for 12.5 g (4.00 mmol) of **L**. The pH in this synthesis was adjusted to 4.5. The addition of 2.50 g (33.53 mmol) of KCl to the synthesis mixture led to red-orange crystals of **5** after three days. The crystals were filtered, dried in desiccators, and recrystallized. The yield was 5.27 g (42.33%). UV: 200, 251 (nm); IR: 3440 vb, 1617 b, 1121 w, 1098 m, 1071 m, 1051 m, 955 vs, 880 m, 796 s, 745 sh, 691 sh, 595, and 506 (cm^−1^); Raman: 979, 890, 519, 235, 156, and 108 (cm^−1^); Analytically calculated (found) (M = 3112.7551): Cu, 2.04 (1.95); K, 7.54 (7.50); P, 1.00 (0.95); V, 1.64 (1.60); W, 59.06 (59.13); and H_2_O, 8.67 (8.73)%.

## 3. Results and Discussions

### 3.1. Determination of Optimal Reaction Conditions for the Synthesis of Complexes ***1–5***

In Figure 1a,b, both the conductivity and spectrometric plots show monolacunary L is stable in the pH range 3.5–6.5, given that the complete structure, [PV_2_W_10_O_40_]^5-^, was performed at a pH under 3.5 [8,12].

In Figure 2, five plots of conductivity variation at different M/L rates (where M is Mn^2+^, Fe^3+^, Co^2+^, Ni^2+^, and Cu^2+^) were recorded. Each plot included two lines, and their intersection point corresponded to the optimal combination rate between M and L (M/L). The first line was assigned by the change in conductivity due to the formation of the complex between M and the ligand L, while the second line was due to the increase in conductivity by adding the metal cation M in excess. In the curves of Figure 2, it is shown that cation M is coordinated to an unit L with a molar report M/L of 1:1, characteristic of monolacunary Keggin ligands [13].

In Figure 3, the plot for each metal cation was traced and the greatest absorbance was found to be at pH 4.5 for the synthesis of the copper complex, respectively, pH 5.0 in others [8]. In Figure 4, it is shown that all the samples have reacted completely after 30 min. All the synthesis reactions of complexes between L and metal cations were took placed better at 60 °C than room temperature (Figure 5), but the absorbance decreased at higher temperatures (between 80 and 100 °C).

### 3.2. Thermogravimetric Study of Ligand L and Complexes ***1–5***

In thermogravimetric analysis the weight loss, recorded in the TG curve between 20 and 320 °C was assigned to the dehydration process. The weight loss of each sample and the corresponding number of water molecules were recorded in Table 1. The weight loss of complexes **1–5** resulted in two stages: in the first stage, the water attributed to crystallization was lost, and in the second stage, the water molecule coordinated in the transition metal cation was lost. In the DTG and DTA curves, it is shown that the loss of water molecules is an endothermic process, resulting in several steps [14,15,16].

The loss of the first water molecules was observed at low temperatures, under 100 °C, due to the efflorescence property specific to this type of compound. The exothermic process in the DTA curve in the range 275–360 °C was caused by the structural damage of polyoxometalates [17]. The final endothermic process in the DTA curve, which was held at a temperature above 460 °C, was due to the oxide formation of atomic components and structural changes resulting from the oxides. Thermal transformations between 700 and 800 °C were due to melting oxides [17].

### 3.3. FT-IR Study

**Figure 6:**
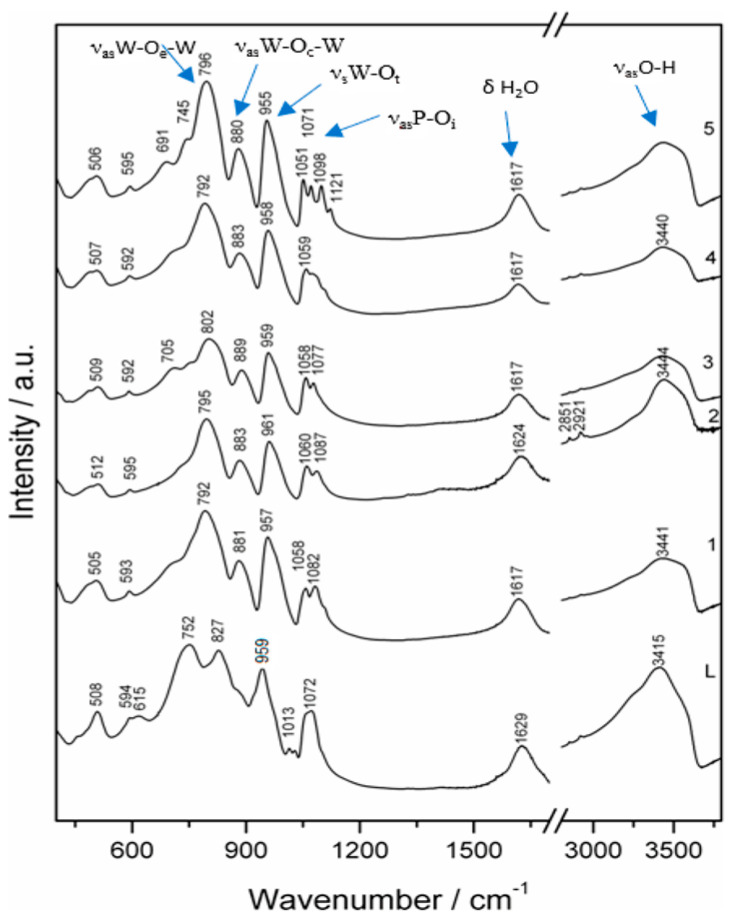
FT-IR spectra of ligand **L** and complexes **1**–**5**.

The IR spectra of complexes **1–5** and monolacunary ligand **L** were plotted in the 4000–400 cm^−1^ range (Figure 6). Specific vibration bands of polyoxometalates structures were obtained from the spectra of monolacunary ligand **L** and complexes **1–5**. Two broad bands from 1600 to 3450 cm^−1^ [18,19] were assigned to vibrations within the crystallization of water molecules and were found in all polyoxometalates spectra, as **L** and complexes **1–5**.

The more intensive vibration band from 3415 cm^−1^ in the spectrum of **L** has shifted to higher energies between 3440 and 3444 cm^−1^ in the spectra of **1–5** and is attributed to the stretching vibration of coordination water involved in hydrogen bonds, the ν_as_(OH) vibration. The medium vibration band from 1629 cm^−1^ in **L** was assigned to δ(OH) in-plane vibration of water molecules involved in hydrogen bonds and shifted between 1617 and 1624 cm^−1^ for complexes **1–5** [15,20,21,22]. The vibration bands between 1000 and 1100 cm^−1^ were assigned to an asymmetrical stretch band (ν_as_(P-O_i_)), where O_i_ is the internal oxygen bond between phosphorus and addenda atoms (tungsten or vanadium) in L, coordinated at transition metal cation in complexes **1–5**, respectively. Monolacunary polyoxometalate L has shown an intense band at 1072 cm^−1^, a weak shoulder around 1100 cm^−1^ and a very weak split band between 1013 and 1040 cm^−1^. In complexes **1–5** vibration bands of P-O_i_ bonds have shifted to higher energies, indicating that the free position of monolacunary structure **L** was occupied by the transitional metal cation M and the symmetry of the polyoxometalate lattice has been partially restored. Two medium intensity bands corresponding to ν_as_(P-O_i_), were recorded between 1051 and 1060 cm^−1^ and between 1071 and 1087 cm^−1^, with a shoulder better defined around 1100 cm^−1^ [20,23]. The most split bands were those for complex **5** of copper, attributed to the Jahn–Teller effect, and the highest symmetry was achieved in complex **4** of nickel. Bands of the stretching vibration ν_as_(Mo-Ot) and ν_as_(V-Ot) (Ot was a terminal oxygen from a M=O bond in each MO_6_ octahedron) appeared as a single intense and sharp band at 943 cm^−1^ in **L** and shifted to a higher energy from 955 to 961 cm^−1^ in spectra for complexes 1–5. The symmetric shape of this vibration band was due to the overlap of the two signals of Mo-Ot and V-Ot bonds [23,24]. In the FT-IR spectrum of **L**, a medium band at 827 cm^−1^ with a shoulder around 880 cm^−1^ was recorded, corresponding to M-Oc-M (M is W^6+^ and V^5+^) bonds, where Oc was an oxygen atom between two corner-sharing MoO_6_ octahedrons [25,26]. This spectrum allure, with splitting of the M-Oc-M band, was due to the decrease in structure symmetry after removing the MO_6_ octahedron from the complete structure. Therefore, M-Oc-M angles located near the lacunary position were modified, and the rigidity and cohesion of anion were reduced. In complexes **1–5**, only one vibrational asymmetric band, ν_as_ (M-Oc-M), has remained and has shifted to higher energies, from 880 cm^−1^ in spectrum **1** to 889 cm^−1^ in spectrum **3** [23,27]. The last intense band from 752 cm^−1^ in spectrum **L** was assigned to M-Oe-M (M is W^6+^ and V^5+^) bonds, where Oe was an oxygen atom between two edge-sharing MoO_6_ octahedrons. The poor delineation from the previous band in the spectrum of **L** was due to two bonds of different lengths. The coordination of the transition metal in complexes **1–5** has restored the polyoxometalate lattice as a single ν_as_ M-Oe-M band and was recorded between 792 and 802 cm^−1^. Both, M-Oc-M and M-Oe-M bonds were better defined and intensive in the complexes **1–5** FT-IR spectra than in the monolacunary **L** spectrum. Moving towards the higher energy of the M-Oc-M and M-Oe-M bands corresponded to a shortening of the bonds, as in the complete structure, characterised by the highest symmetry and structural stability of (between) Keggin polyoxometalates, which proved that oxygen atoms (Oc and Oe) were involved in coordination of a transition metal cation, M [27]. The band positions in the spectra of compounds 1–5 showed an increase in polyoxometalate structural symmetry compared to the monolacunary ligand **L** [27].

### 3.4. Raman Study

**Figure 7:**
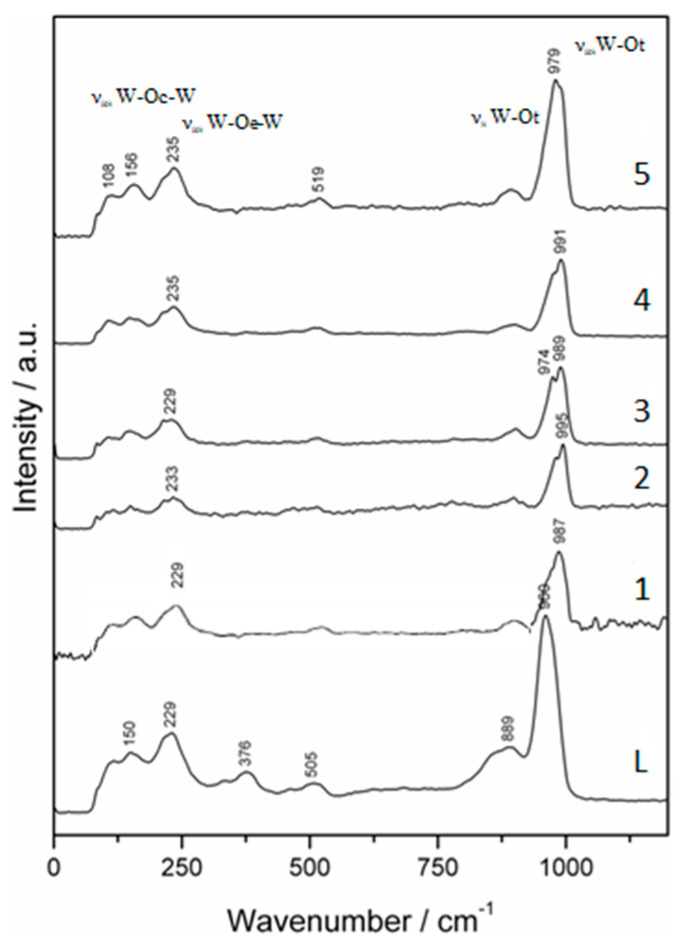
The Raman spectra of ligand **L** and complexes **1**–**5**.

The Raman spectra of complexes **1–5** and monolacunary Keggin ligand **L** are shown in Figure 7, in the 1200–100 cm^−1^ range.

In the Raman spectrum of the ligand **L**, both bands, one intensive at 960 cm^−1^ and the other weaker at 889 cm^−1^, can be assigned to W-Ot symmetric and asymmetric stretching vibrations. Instead, in the spectra for complexes **1–5**, the W-Ot stretching vibration represented an intensive band with a shoulder shifting to higher energies from 979 cm^−1^ for **5** to 995 cm^−1^ for **2**, compared to 960 cm^−1^ for **L**. The W-Ot band of ligand **L** of 889 cm^−1^ was observed to be weaker in the spectra for complexes **1–5** because the complete Keggin lattice was rebuilt after coordination of the metal cation [23,28]. In spectrum **L**, the M-Oc-M and M-Oe-M bonds (M is W^6+^, V^5+^) were assigned to a band of 229 cm^−1^, which in spectra **1–5** has shifted slightly to higher energies (235 cm^−1^ maximum in spectra **4** and **5**). The broad Raman bands from 505 cm^−1^ and the less intense vibrations from 150 cm^−1^ and 376 cm^−1^, are due to the symmetric and asymmetric M-Oi-M bonds (M = W^6+^ and V^5+^). In the monolacunary structure **L** the spectra of complexes **1–5** became weaker [21,29,30].

### 3.5. UV-VIS Study

**Figure 8:**
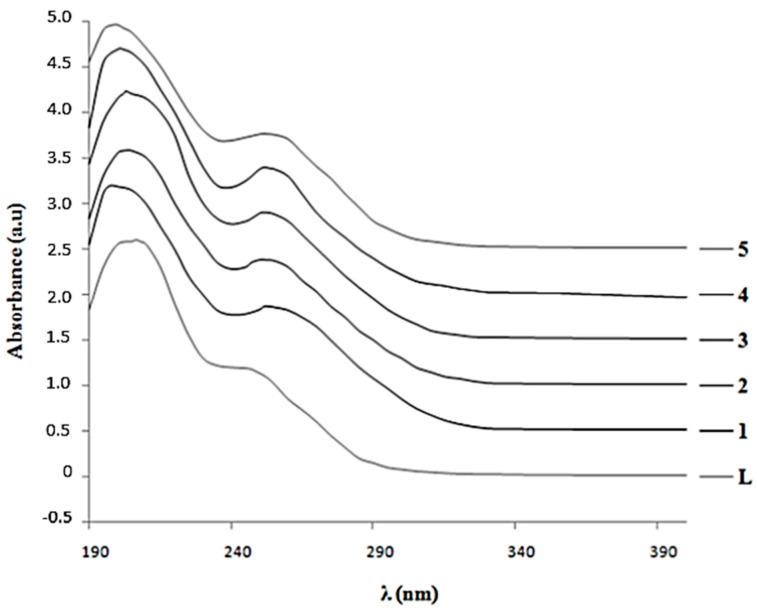
The UV spectra of ligand **L** and complexes **1**–**5**.

The UV spectra of ligand **L** and complexes **1–5** are represented in Figure 8, where two charge-transfer (CT) bands characteristic of all polyoxometalates can be observed. The most intense band can be assigned to dπ-pπ transitions corresponding to W=O_t_ and V=O_t_ bonds (ν_2_), which appeared at 48,430 cm^−1^/206.5 nm in spectrum **L**.

This band shifted at higher energies in the spectra of complexes **1–5** to 50,250 cm^−1^/199 nm for **1**, 49,020 cm^−1^/204 nm for **2**, 492,610 cm^−1^/203 nm for **3**, 49,710 cm^−1^/201 nm for **4**, and 50,030 cm^−1^/200 nm for **5**. The second band (ν_1_) was assigned to dπ–pπ-dπ transitions from M-Oc-M and M-Oe-M bonds (M is W^6+^ and V^5+^), and it was less defined and shifted to higher intensities, 40,160 cm^−1^/249 nm in spectrum **L** than in similar spectra of complete Keggin structures [15,21,31]. This shift was due to the asymmetry created by the presence of the vanadium atom in the structure and the lacunary position. In the spectra of complexes **1–5**, the ν_1_ band became more intensive and better defined than in **L** after the linking of metal cations in the lacunary position and was shifted toward lower energies of 39,760 cm^−1^/251.5 nm for **1**, 40,020 cm^−1^/250 nm for **2**, 39,840 cm^−1^/251 nm for **3**, 39,680 cm^−1^/252 nm for **4**, and 39,760 cm^−1^/251.5 nm for **5**. The position and shape of bands in spectra **1–5** resemble those with complete Keggin structures, which indicates the formation of a Keggin lattice after coordination of the metal cations (Mn^2+^, Fe^3+^, Co^2+^, Ni^2+^, and Cu^2+^) [20,29,32,33,34].

The electronic bands of the d-d transition characteristic for metal cations coordinated by a monolacunary polyoxometalate ligand, **L**, can be found in the VIS spectra. Only d-d transition bands of Co^2+^, Ni^2+^, and Cu^2+^ can be determined in the VIS spectra because intense bands of Mn^2+^ and Fe^3+^ ions are covered by bands of **L** [35]. Solutions of monolacunary **L** and its complexes **1–5** have an orange colour and exhibit a very intensive band in VIS at 468 nm/21,370 cm^−1^. This visible band may be due to a charge transfer transition of oxygen to addenda atoms (O→M) (M = W^6+^ and V^5+^). Both curves of complexes and aqua cations were very similar, corresponding to the preservation of an octahedral coordination of metal cations in complex molecules. The coordination of metal cations to polyoxoanions shifted the cation transition band to lower energies than in aqua complexes, due to weaker interactions between the metal cation and the oxygen atoms of polyoxoanions.

**Figure 9:**
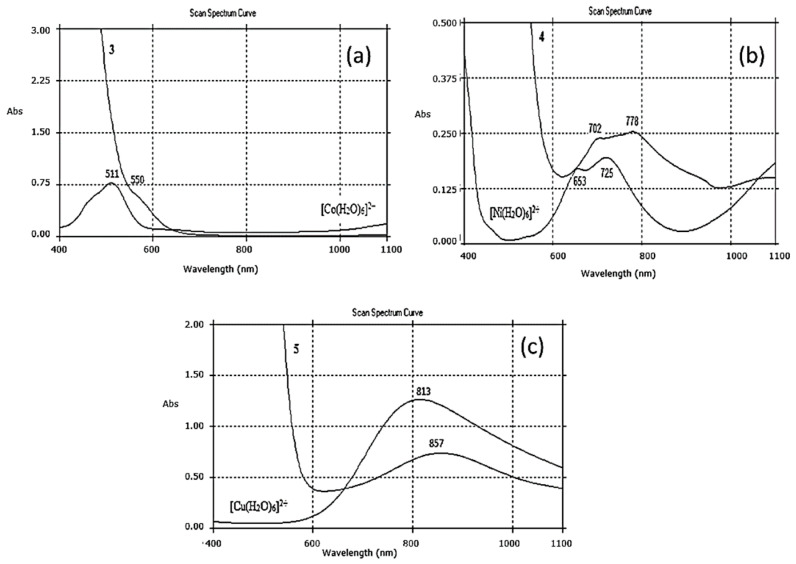
The VIS spectra of (**a**) [Co(H_2_O)_6_]^2+^ and complex **3;** (**b**) Ni(H_2_O)_6_]^2+^ and complex **4;** and (**c**) Cu(H_2_O)_6_]^2+^ and complex **5**.

The characteristic band of the cation [Co(H_2_O)_6_]^2+^ from 511 nm/19,570 cm^−1^ corresponding to an energetic transition ^4^T_1g_(P) ← ^4^T_1g_ (F), (ν_2_) has shifted to lower energies in complex **3**, and appears at 550 nm/18,180 cm^−1^, where it overlaps with the intense band of complex **3** in the VIS spectrum (Figure 9a).

This shift was due to the decrease in the symmetry of the octahedral ligand fields of Co^2+^ in complex **3** compared to the aqua ion, [Co(H_2_O)_6_]^2+^ [36].

In complex **4**, the Ni^2+^ cation had a d^8^ electronic configuration with the fundamental term ^3^F_4_. The Ni^2+^ cation occupied a central position in the NiO_6_ octahedron after coordination by monolacunary polyoxometalate, **L**. An hydrated ion [Ni(H_2_O)_6_]^2+^ with an octahedral configuration, coordinated by six oxygen atoms as in complex **4**, was chosen as the reference. The VIS absorption spectra of complex **4** and the aqua cation [Ni(H_2_O)_6_]^2+^ are shown in Figure 9b.

For Ni^2+^ in an octahedral field, it was expected to see three d-d electronic bands: ^3^A_2g_(F) → ^3^T_2g_(F) = (ν_1_) ~ 8700 cm^−1^/1150 nm; ^3^A_2g_(F) → ^3^T_1g_(F) = (ν_2_) ~ 14,500 cm^−1^/689 nm, and ^3^A_2g_(F) → ^3^T_1g_(P) = (ν_3_) ~ 25,300 cm^−1^/395 nm. Charge-transfer bands due to a high intensity can mask some d-d bands, so that in the spectra only one or two bands can be seen. Moreover, band ν_2_ is generally of very low intensity. The allure of the VIS electronic spectra of complex **4** it is characteristic for octahedral symmetry (O_h_), as in the hydrated ion [Ni(H_2_O)_6_]^2+^. Only a low band, ν_2_ to 713 nm/14,025 cm^−1^, was observed in the spectrum for complex **4** and was shifted to lower energies than in the [Ni(H_2_O)_6_]^2+^ spectrum (653 nm/15,340 cm^−1^). This movement of band ν_2_ indicated that the Ni^2+^ cation has a weaker coordination by oxygen atoms of the monolacunary polyoxometalate **L** than water molecules in [Ni(H_2_O)_6_]^2+^. The NiO_6_ octahedron in complex **4** was distorted due to non-equivalent bonds. Both bands from 798 nm/12,530 cm^−1^ in the spectrum of complex **4** and the 725 nm/13,800 cm^−1^ band in the spectrum of [Ni(H_2_O)_6_]^2+^ were assigned to the spin forbidden transition ^3^A_2g_(F) →^1^E_g_(D), and the intensity of this transition increased on account of the intensity theft from the allowed electronic transition. The band (ν_1_) from the near IR was not visible in both **4** and [Ni(H_2_O)_6_]^2+^ because the spectrophotometer was limited to measurements in the 190–1100 nm range.

The VIS spectrum of complex **5** (Figure 9c) presents a broad absorption with a maximum around 857 nm/11,650 cm^−1^, suggesting a distorted octahedral environment around the Cu^2+^ (d^9^) cation.

This band was attributed to the ^2^T_2g_(D)← ^2^E_g_(D) (ν_1_) transition and shifted to a lower energy in complex **5** than in [Cu(H_2_O)_6_]^2+^ (813 nm/12,300 cm^−1^) because of the Jahn–Teller effect, characteristic of a copper ion coordinated with different ligands, in this case a water molecule and different oxygen atoms of **L**.

### 3.6. Powder XRD Study

**Figure 10:**
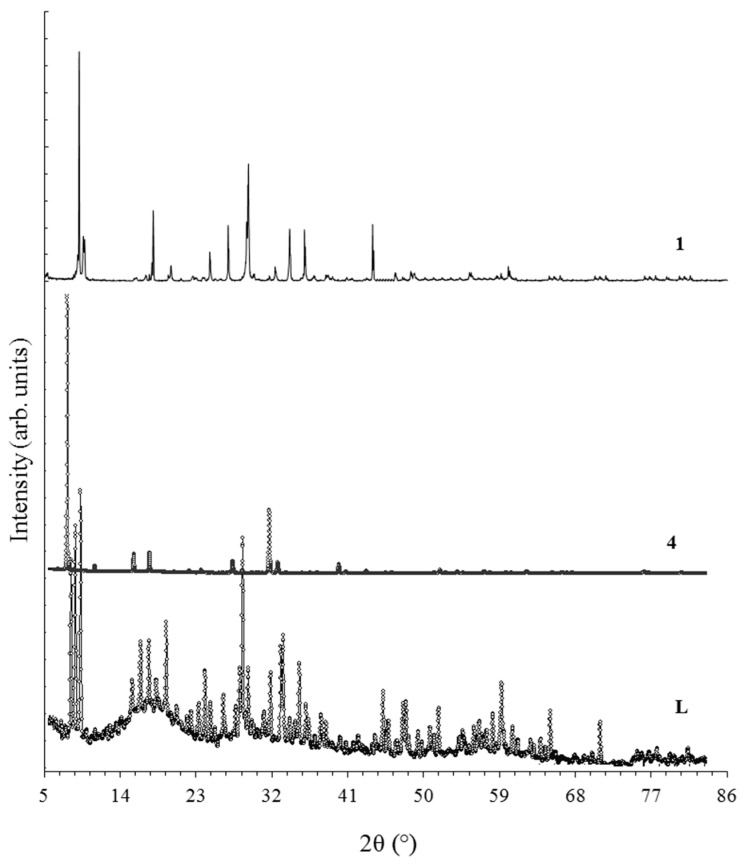
XRD spectra of ligand **L** and complexes **1** and **4**.

The patterns indexed using the Dicvol method powder XRD pattern for **L** and complexes **1** and **4** are presented in Figure 10. Based on the calculation and determination of Miller indices the following parameters for monolacunary ligand L crystallization in a tetragonal system were determined: a = b = 19.888 Å and c = 24.729 Å (α = β = γ = 90°) was determined, with a unit cell volume V = 9781 Å^3^ and density, D_calc_ (D_exp_) = 4.2468 (4.2340) g cm^−3^.

Both complexes **1** and **4** were found to have a primitive cubic structure, assigned to the Pm-3n space group. A primitive cubic structure is expected to have the entire studied group of complexes of the same Keggin ligand, **L**. The cell parameters of **1** were a = b = c = 21.636 Å (α = β = γ = 90°), with a volume V = 10,633.5 Å^3^ and density, D_calc_ (D_exp_) = 3.8774 (3.8658) g cm^−3^, while for **4**, a = b = c = 22.191 Å (α = β = γ = 90°) was found, with a volume V = 10,928 Å^3^ and density, D_calc_ (D_exp_) = 3.8774 (3.8658) g cm^−3^. The increase in cell parameters for complex **4** compared to complex **1** is due to the increasing number of crystallisation water molecules in lattice **4** [31,37,38,39]. For complexes **1** and **4**, the first diffraction peak was very intense compared to the others because crystallites were oriented preferentially in the sample [27,40]. The average crystallite size D for samples **L**, **1**, and **4** was calculated using the Debye–Scherrer formula [41]. We have obtained the following values: 740 Å for **L**, 1400 Å for **1**, and 1500 Å for **4**. The degree of crystallinity, Xc, was evaluated as the ratio of the area due to the diffraction peaks and the total diffraction area, which includes diffraction peaks and an amorphous halo. To assess the degree of crystallinity, the Reflex computer program, part of the Material Studio software suite, was used. The results obtained for the degree of crystallinity were: 55% for **L**, 93% for **1**, and 94% for **4**. One can see that the degree of crystallinity is correlated with crystallite size, samples **1**–**4** being very crystalline while the crystallinity of the polyoxometalate ligand **L** is moderate. The coordination of the Mn^2+^ or Ni^2+^ cations in a lacunary position for **L** contributed to the stabilization of the crystal lattice and the increase of symmetry from a tetragonal to a cubic system [42]. For each of the studied compounds, the number of molecules in a unit cell is 8.00, while the experimental parameter Z is 7.98 [43].

## 4. Conclusions

The conductometric method showed that the complexes of the monolacunary Keggin ligand K_8_[PVW_10_O_39_]·15H_2_O with transition metal cations have the general formula K_n_[MPVW_10_O_39_(H_2_O)]·xH_2_O, similar to complexes already mentioned in the literature [5]. The optimal pH for synthesis was found to be a value of 4.5 for the copper complex and 5.0 for the other complexes, which is characteristic for Keggin tungstophosphate, even when partially substituted with molybdenum addenda [2,8].

The IR spectra showed that the transition metal cation was coordinated in a lacunary position in K_8_[PVW_10_O_39_]·15H_2_O lattice, involving oxygen atoms from M-Oe-M, M-Oc-M, and P-Oi bonds, as in other complexes of monolacunary Keggin ligands [20,23,24,25,26,27]. 

IR, Raman, and UV spectra revealed that K_n_[MPVW_10_O_39_(H_2_O)]·xH_2_O complexes have higher stability and symmetry than monolacunary K_8_[PVW_10_O_39_]·15H_2_O after coordination of a transition metal cation [28,29,35]. The VIS data of polyoxometalate complexes revealed that Co^2+^, Ni^2+^, and Cu^2+^ cations were coordinated in an octahedral ligand field, and coordination was performed with oxygen atoms. For each metal cation Co^2+^, Ni^2+^, or Cu^2+^, an octahedral ligand field created by monolacunary polyoxometalate was weaker compared to water molecules in the aqua ions of a transition metal cation [36]. Based from recorder X-ray spectra, can conclude that complex K_6_[MnPVW_10_O_39_(H_2_O)]·14H_2_O and K_6_[NiPVW_10_O_39_(H_2_O)]·19H_2_O have high crystallinity, being crystallised in a cubic system, while monolacunary polyoxometalate K_8_[PVW_10_O_39_]·15H_2_O presented moderate crystallinity due to high structural instability, which was associated with a lacunary position in the polyoxometalate lattice [37,40].

Another Keggin compound with a similar structure as **L** and **1–5** complexes has been researched for its versatile properties, like good solubility in organic solvents and water, easy counter-ion exchange, and reversible redox properties. Their potential has not been completely explored [44]. For example, the recently, catalytic properties of different [PW_12-x_V_x_]^n-^ anions proved to be interesting, especially for the oxidation of organic compounds [45,46,47]. Additionally, Keggin compounds with different compositions are recommended to be used in rechargeable batteries [46] or to fabricate mesoporous materials [48,49].

## Figures and Tables

**Table 1:** Thermogravimetric data of ligand **L** and complexes **1–5**.

Thermogravimetric Process		L	1	2	3	4	5
DTA (°C)	Endothermic	62	80.5	75.5	83	88	71
			107	113.5	118	178	164
	Exothermic	275	344.5	344.5	356	360	339.5
	Endothermic	500	457.5	460.5	468	479	540
			500	550	521	546.5	
DTG (°C)		54.5	77	68.5	79	80.5	67
		98.5	155	106	111	117.5	104.5
		154.5	350	163	167	173.5	158.5
		295		339	350.5		332
TG	Weight lost (%)	8.69	8.78	6.83	12.15	10.65	7.76
			0.58	0.62	0.58	0.56	0.97
	H_2_O no. molecules	15	14	11	21	19	14
			1	1	1	1	1

## Data Availability

Not applicable.
